# The Protective Effect of *E. faecium* on *S. typhimurium* Infection Induced Damage to Intestinal Mucosa

**DOI:** 10.3389/fvets.2021.740424

**Published:** 2021-10-15

**Authors:** Hang Zhang, Minjuan Wang, Junpeng Jia, Jiayi Zhao, Stoffel Matjeke Radebe, Qinghua Yu

**Affiliations:** Ministry of Education (MOE) Joint International Research Laboratory of Animal Health and Food Safety, College of Veterinary Medicine, Nanjing Agricultural University, Nanjing, China

**Keywords:** probiotics, intestinal mucosa, salmonella, proliferation, Wnt/β-catenin signal pathway

## Abstract

Intensive farming is prone to induce large-scale outbreaks of infectious diseases, with increasing use of antibiotics, which deviate from the demand of organic farming. The high mortality rate of chickens infected with *Salmonella* caused huge economic losses; therefore, the promising safe prevention and treatment measures of *Salmonella* are in urgent need, such as probiotics. Probiotics are becoming an ideal alternative treatment option besides antibiotics, but the effective chicken probiotic strains with clear protective mechanism against *Salmonella* remain unclear. In this study, we found *Enterococcus faecium* YQH2 was effective in preventing *Salmonella typhimurium* infection in chickens. *Salmonella typhimurium* induced the loss of body weight, and liver and intestinal morphology damage. The inflammatory factor levels increased and intestinal proliferation inhibited. However, after treatment with *Enterococcus faecium* YQH2, broilers grew normally, the pathological changes of liver and intestine were reduced, and the colonization of *Salmonella* in the intestine was improved. Not only that, the length of villi and the depth of crypts were relatively normal, and the levels of inflammatory factors such as IL-1β, TNF-α, and IL-8 were reduced. The number of PCNA cells of *Enterococcus faecium* YQH2 returned to normal under the action of *Salmonella typhimurium* infection, which was conducive to the normal proliferation of intestinal epithelial cells. The protective effect of *Enterococcus faecium* YQH2 may be due to the attribution to the activation of hypoxia and then induced the proliferation of intestinal stem cells to repair the damage of intestinal mucosa under *Salmonella typhimurium* infection. This study demonstrated that *Enterococcus faecium* YQH2 was effective in preventing *Salmonella typhimurium* infection, which could be further used in the chicken health breeding.

## Introduction

With the improvement of people's living standards and the increase in demand for livestock and poultry products, strict health cultivation of chickens shows a sharp increase. Chicken has a lower fat and higher protein ratio as compared with other meats; therefore, it is popular all over the world as a healthier option for lean protein meat (1). In recent years, with the rapid development of the livestock and poultry breeding industry, the diseases acquired by livestock and poultry have also shown an increasing trend year by year (2). Bacterial infectious diseases have become an important element restricting the development of many country's agriculture industry. *Salmonella* is also often present in the intestines of many farm animals, especially in chickens (3, 4). Salmonellosis can cause illnesses in both humans and animals, and it is highly contagious. There is the evidence that people are susceptible to intestinal infections if they ate large amounts of chickens contaminated with *Salmonella* or come into direct contact with sick chickens (5, 6). *Salmonella* can be roughly divided into *Salmonella typhi* and *non-Salmonella typhi*. *Salmonella typhimurium* belonged to *Salmonella Paratyphi*. Compared with other serovars, *Salmonella typhimurium* (*S. typhimurium*) was known to cause severe gastro-intestinal inflammation and even death, which has caused great harm to the agriculture industry (7, 8). Therefore, we need to find effective ways to strengthen the prevention, diagnosis, and treatment of salmonellosis.

In recent years, with the extensive use of antibiotics, *Salmonella typhimurium* has developed resistance to existing antibiotics, and the treatment effect was extremely poor. Therefore, it is necessary to find new treatments to prevent *S. typhimurium* infections (9). Probiotics have entered people's field of vision with the advantages of safety and no residue, and are considered to promote the growth of animal husbandry. Probiotics are already used in livestock breeding, and could significantly improve the immune function of chickens, improve the intestinal barrier, and alleviate oxidative stress (10–12). In poultry farming, there is an urgent need for probiotics that will enhance the early life development and protects the well-being of broilers. Due to the evidence of host and strain specificity of probiotic function, the probiotics with much greater specificity are needed in chickens. *Enterococcus faecium*, as a kind of probiotics, is considered safe and has strong intestinal adhesion and colonization ability, and can also inhibit the growth of harmful microorganisms in the intestines (13). Studies have shown that adding *Enterococcus faecium* to broiler chicken feed could significantly improve the growth performance of broiler chickens and promote the digestion and absorption of nutrients (14). *Enterococcus faecium* YQH2 (*E. faecium* YQH2) is a gram-positive enterococcus that is a symbiont (innocuous, coexisting organism) in the gastrointestinal tract of humans and animals (15). A research study showed that *E. faecium* NCIMB 10415 have reduced the pathogenic bacterial load in healthy piglets (16). Previous studies demonstrated that *E. faecium* has good therapeutic effect on S. *typhimurium* infection in pigs (17). The effect of *Enterococcus faecium* on *Salmonella typhimurium* is less studied. Recent studies have confirmed that adding *Enterococcus faecium* NCIMB11181 in feed can improve feed conversion efficiency and reduce *Salmonella* colonization in the intestine (18). However, the specific research mechanism of *Enterococcus faecium* against *Salmonella typhimurium* is still unclear.

*S. typhimurium* infection is also accompanied by a serious intestinal inflammation and intestinal mucosal damage (19). The proliferation of intestinal stem cell, modulated by the Wnt/β-catenin signal pathway (20), is necessary to repair damaged epithelial cells and alleviate intestinal inflammation. Hypoxia-inducible factors (HIF) played a very important role in the intestinal epithelial barrier and maintain the homeostasis of the intestinal environment (21). A previous study reported that hypoxia could inhibit Wnt pathways and hypoxia-inducible factor-1 alpha (HIF-1α) inhibited the hARD1-mediated β-catenin activation to obstruct the Wnt signaling pathway (22). Moreover, HIF-1α could also inhibit the expression of TLR4 and transcription factors NF-κB, thereby ameliorating inflammation (23). Nevertheless, the relationship between *Enterococcus faecium* and hypoxia-inducible factors and the specific mechanism of how the two together protect the intestinal mucosal barrier have not yet been fully elucidated. Therefore, this study mainly explored the detailed protective mechanism of *Enterococcus faecium* YQH2 against *Salmonella typhimurium* infection in chickens, from the sides of intestinal epithelial proliferation and hypoxia-inducible factors.

## Materials and Methods

### Animal Experiments and Bacteria

**Figure 1:**
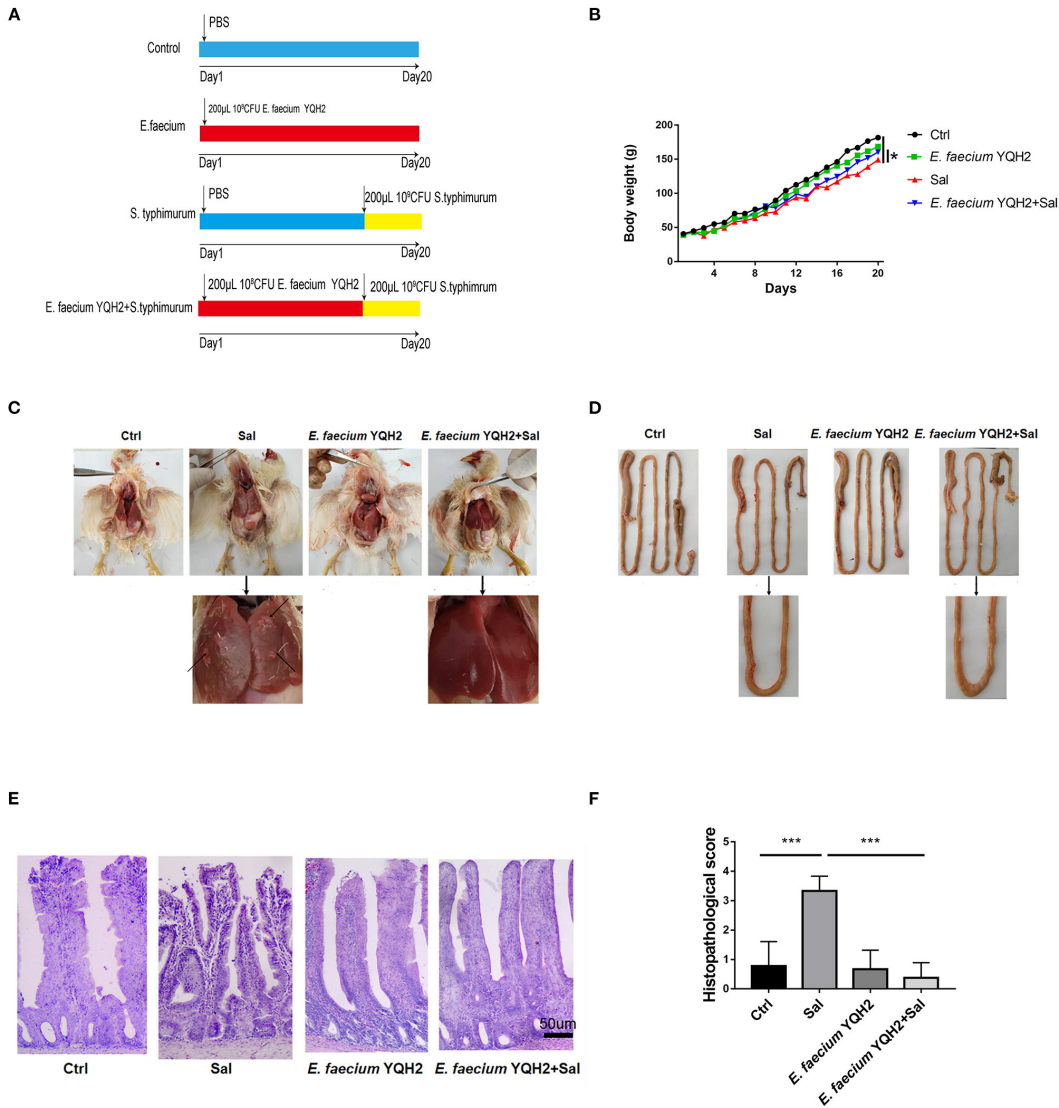
*Enterococcus faecium* YQH2 obviously alleviated the histopathologic changes caused by *S. typhimurium*. **(A)** Broilers were orally administered with 200 μl PBS in control group or 200 μl 10^8^ CFU of *E. faecium* in YQH2 group for a consecutive period of 20 days. Chickens of *S. typhimurium* group were orally administered with 10^9^ CFU *S. typhimurium* on the 14th day. Chickens of YQH2+*S*. *typhimurium* group were orally administered with 10^8^ CFU *E. faecium* for 20 consecutive days and orally administered with 10^9^ CFU *S. typhimurium* on the 14th day. **(B)** Changes in body weight of each group of broilers within 20 days, *n* = 8. **(C,D)** Apparent pathological changes in the liver and intestinal of chickens in four groups. **(E)** H&E staining of ileums in different groups (scale bar = 50 μm), *n* = 6. **(F)** The histopathological score of the ileum in different groups according to the reference standard of pathology score, *n* = 6. *p < 0.05, **p < 0.01, ***p < 0.001.

A total of thirty-two 1-day-old white feather broilers were randomly divided into four groups: controls (orally administered with PBS for a period of consecutive 20 days, *n* = 8), YQH2 group (orally administered with 10^8^ CFU *E. faecium* for consecutive 20 days, *n* = 8), Sal (orally administered with 10^9^ CFU *S. typhimurium* on the 14th day), and YQH2+Sal (orally administered with 10^8^ CFU *E. faecium* for consecutive 20 days and orally administered with 10^9^ CFU *S. typhimurium* on the 14th day). The related experimental animal design is described in Figure 1A. All chickens drank and ate freely, and were not fed antibiotics during the experiments. The relative humidity in the room was 45–60%. The animal studies were approved by the Institutional Animal Care and Use Committee of Nanjing Agricultural University.

*Enterococcus faecium* YQH2 was isolated from broiler feces. *Salmonella typhimurium* was saved in the laboratory and has streptomycin resistance. *S. typhimurium* was grown at 37°C in Luria–Bertani (LB) broth (10 g tryptone, 5 g yeast extract, and 5 g NaCl per liter) or on LB plates fortified with 1.5% agar for 24 h with shaking.

### The Body Weight and Sample Collection

The body weight of broilers in all groups were weighed and recorded every 3 days. On the 21st day, broilers were sacrificed to remove their ileum and liver to observe. Feces of six chickens in each group were collected. Their ileum fragment samples were collected from each group, respectively, and stored at −20°C for further detection or fixed in the 4% neutral buffered formalin for at least 24 h and then embedded in paraffin. Fecal samples were stored at −70°C for subsequent experiments. For molecular experiments, the remaining sections of ileum samples were stored in −70°C.

### *S. typhimurium* Colonization in Chicken Ileum

Broiler fecal samples were taken from −70°C to observe the colonization of *Salmonella typhimurium*. In brief, each group of 0.2 g fecal samples was weighed, and then 1 ml of sterile PBS was added to shake and mix. Diluted by gradient with sterile PBS, the dilution gradients were 10^−1^, 10^−2^, 10^−3^, 10^−4^, 10^−5^, and 10^−6^. Then 10^−5^ and 10^−6^ gradients of liquid were selected, and 100 μl of each gradient in each group were evenly spread on 1.5% LB agar plates and grown at 37°C for 36 h. Each LB plate contained 50 μg/ml streptomycin.

### Observation of Intestinal Morphology and Histopathological Evaluation

The collected ileum samples were taken out of paraformaldehyde and used for HE staining. In short, after a series of dehydration, transparency, wax penetration, and embedding, the ileum tissue was sliced into continuous slices with thickness of 5 μm, stained with H&E, and examined under an Olympus optical microscope. The length of ileum villi was measured by ImageJ software. Histopathology was detected under a microscope. The ileum tissues of each group were scored by blind method according to the histopathological scoring reference standards (24). In short, three indexes closely related to the degree of inflammation were tested (0–4: none, mild, moderate, severe, all), mucosal injury (0–4: none, mucosal, mucosal and submucosal, transmural, all), crypt damage (0–4: none, basal one-third damaged, basal two-thirds damaged, entire crypt lost, entire crypt, and epithelium lost). The score statistics method was to multiply the score of each parameter by the percentage that reflected the degree of tissue damage (×1, 0–25%; ×2, 26–50%; ×3, 51–75%; ×4, 76–100%), add up all the scores, and take the average of each group; the highest score was 4. Each group was guaranteed to count the intestinal morphology under 30 fields of view.

### Immumohistochemistry Assay

The ileum tissue sections were immersed in xylene twice then placed in ethanol and water in turn. The slides were placed in a beaker containing citrate buffer solution and performed antigen retrieval at 98°C for 20 min. After the slide has cooled down at room temperature, the tissue was neutralized with 3% endogenous peroxidase solution for 10 min and sealed the tissue with 5% bovine serum albumin for 1 h at room temperature. Diluted anti-chicken PCNA (1:100) antibody was added and incubated at 4°C overnight. Then a second antibody was added and incubated at 37°C for 1 h. The tissues were incubated with horseradish peroxidase for 1 h at room temperature. DAB was added to react color. The tissues were sealed with neutral resin glue. The PCNA cell images were observed under the microscope and collected. Image-Pro Plus software was used to calculate and analyze the distribution of PCNA cells in crypts.

### RNA Extraction and Quantitative Real-Time PCR in Tissues

The total RNA in the ileum was extracted with RNAiso Plus (Takara). After the RNA was extracted, the concentration was calculated and 1 μg of total RNA was reacted with PrimeScript RT Reagent Kit according to the manufacturer's instructions (Takara). Reverse transcription of the RNA was performed with the reagents. The cDNA was used for qRT-PCR using SYBR Green Master Mix (Takara) and Q7 (Life) with the primers listed in Table 1. GAPDH gene was used as an internal reference gene to quantify the expression level of other genes. The qPCR thermal cycling conditions were as follows: 5 min at 95°C, followed by 40 cycles of 15 s at 95°C and 34 s at 60°C. All samples were run in duplicate, and gene expression levels were analyzed using the 2^−ΔΔCt^ method.

**Table 1:** Primers for real-time PCR.

**Target gene**	**Primer sense (5^′^-3^′^)**	**Primer antisense (5^′^-3^′^)**
VEGFA	CGATGAGGGCCTAGAATGTGTC	AGCTCATGTGCGCTATGTGC
VEGFR1	TGTAACTAAGTATGCCTGTGG	GGAGTTGTTGGGTATCTGC
Wnt3	GAAGCTGCGAGGTCAA GACT	TTGCACGTTCTGTCCCTTGT
β-Catenin	CGCCATTTTAAGCCTCTCGC	CCTTTCAGAGACTGTGGCACG
Lrp5	TACTGGGTAGATGGGCGTCA	GGTCATGTGGCTGCTTCTCT
HIF-1α	TGAGAGAAATGCTTACACACAG	TGATGGGTGAGGAATTGGTTCAC
HIF-2α	AGCAGTGCCTTTGAGAATGG	TGCACTTGGAGTACTGCTGC
TLR-4	CTGCAGTTTCTGGATCTTTCAA	TAAGCCATGGAAGGCTGCTA
GAPDH	TGATGGTCCACATGGCATCC	GGGAACAGAACTGGCCTCTC
IL-1β	TGCCTGCAGAAGAAGCCTCG	GACGGGCTCAAAACCTCCT
TNF-α	CAGATGGGAAGGGAATGAAC	CACACGACAGCCAAGTCAAC
IL-8	GTGAACGGCAACTCTTGGA	GCAGTGGGGCCGCTTGG
MyD88	CTGGCATCTTCTGAGTAGT	TTCCTATGTTCTGGCTTCT
NF-KB	CTGAAACTAGGATTGCTGCGGA	GCTATGTGAAGACTGCGTTGTGC
P50	GGAAAGTCACAGAACCACAGAA	CCAGCAGCATCTTCACATCTC

### Statistical Analysis

The results were expressed as the means ± SD. One-way ANOVA was employed to identify significant differences among multiple groups. ^*^p < 0.05, ^**^p < 0.01, and ^***^p < 0.001. Data were combined from at least three independent experiments unless otherwise stated.

## Results

### *Enterococcus faecium* YQH2 Protected Chicken From *Salmonella typhimurium*–Induced Enteritis

To detect the protective effect of *Enterococcus faecium*, we first continuously detected the weight change of broilers within 20 days. The results showed that chickens fed with *Enterococcus faecium* alone did not promote weight gain. However, compared with chickens infected with *Salmonella*, the weight of chickens treated with *E. faecium* YQH2 for 20 consecutive days returned to normal (Figures 1A,B). *S. typhimurium*–induced enteritis showed characteristic intestinal pathoanatomical symptoms with intestinal and liver (Figures 1C,D). The liver showed mononuclear cell infiltration and diffused areas of white focal necrosis after *S. typhimurium* infection. Beyond that, intestinal hemorrhage and shortening caused by *S. typhimurium* could also be observed. To further observe the inflammatory changes caused by *S. typhimurium*, H&E staining was used to help observe microscopic symptoms (Figure 1E). The intestinal structural integrity was compromised with destroyed morphological structures, shortened length of villi, and hemorrhagic spot on the top of villi by *S. typhimurium* compared with normal chicken. However, *Salmonella typhimurium*–infected chickens treated with *E. faecium* YQH2 reduced ileal bleeding points and significantly improved liver, intestinal morphology, and villus length. In addition, the histopathological score of the ileum also showed that *E. faecium* YQH2 had a lower score under the condition of *Salmonella typhimurium* infection, indicating that *Enterococcus faecium* has an antagonistic effect on *Salmonella typhimurium* (Figure 1F). Together, these results showed that *Enterococcus faecium* had a protective effect on broiler chickens infected with *Salmonella*.

### *Enterococcus faecium* YQH2 Improved Intestinal Inflammation Caused by *Salmonella typhimurium*

**Figure 2:**
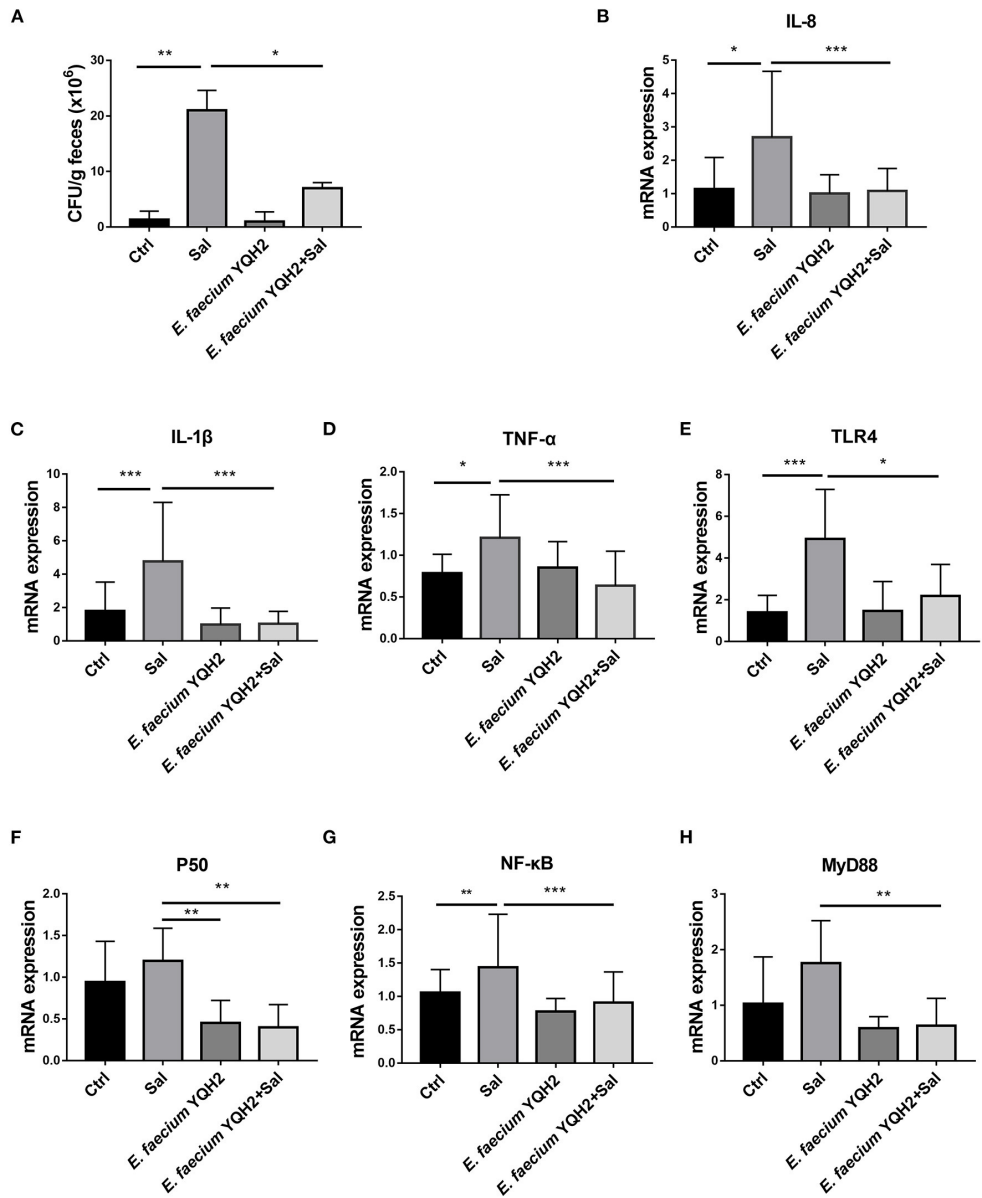
*Enterococcus faecium* YQH2 downregulated the NF-κB signaling pathway to improve the inflammation. **(A)** The level of *S. typhimurium* in feces was detected by colonization test. **(B–H)** The mRNA expressions of IL-8, IL-1β, TNF-α, TLR4, P50, NF-κB, and MyD88 detected by RT-qPCR, *n* = 8. *p < 0.05, **p < 0.01, ***p < 0.001.

Then, we calculated the load of *Salmonella typhimurium* in the ileum. The statistical results showed that the colonization of *Salmonella typhimurium* in the feces of *Salmonella typhimurium*–infected chickens reached 2 × 10^7^ CFU/g, while the colonization of *Salmonella typhimurium* in the ileum after the treatment of *E. faecium* YQH2 was significantly reduced, about three times lower (Figure 2A). Furthermore, *S. typhimurium* infection significantly increased the inflammatory cytokine levels of IL-8, IL-1β, and TNF-α compared with the control group (*p* < 0.05), which were also reduced with the pre-treatment of *E. faecium* YQH2 (*p* < 0.01), consistent with the *S. typhimurium* colonization (Figures 2B–D). TLR/Myd88/NF-κB is an important inflammation signaling pathway in the body. TLR can activate Myd88, which in turn leads to the activation of the NF-κB signaling pathway, which ultimately leads to inflammation. After *Salmonella typhimurium* was infected, the expression of these signaling pathway factors related to inflammation increases, which was consistent with the previously observed H&E staining results (Figure 1E). However, *E. faecium* YQH2 also maintained normal mRNA expression of TLR4, P50, NF-κB, and Myd88 both under physiological status and *S. typhimurium–*induced pathological status (Figures 2E–H). In general, *Salmonella typhimurium* could cause an increase in inflammatory factors, but *E. faecium YQH2* improved intestinal inflammation caused by *Salmonella typhimurium*.

### *Enterococcus faecium* YQH2 Promoted Intestinal Stem Cell Proliferation by Activation of Wnt/β-Catenin Pathway

**Figure 3:**
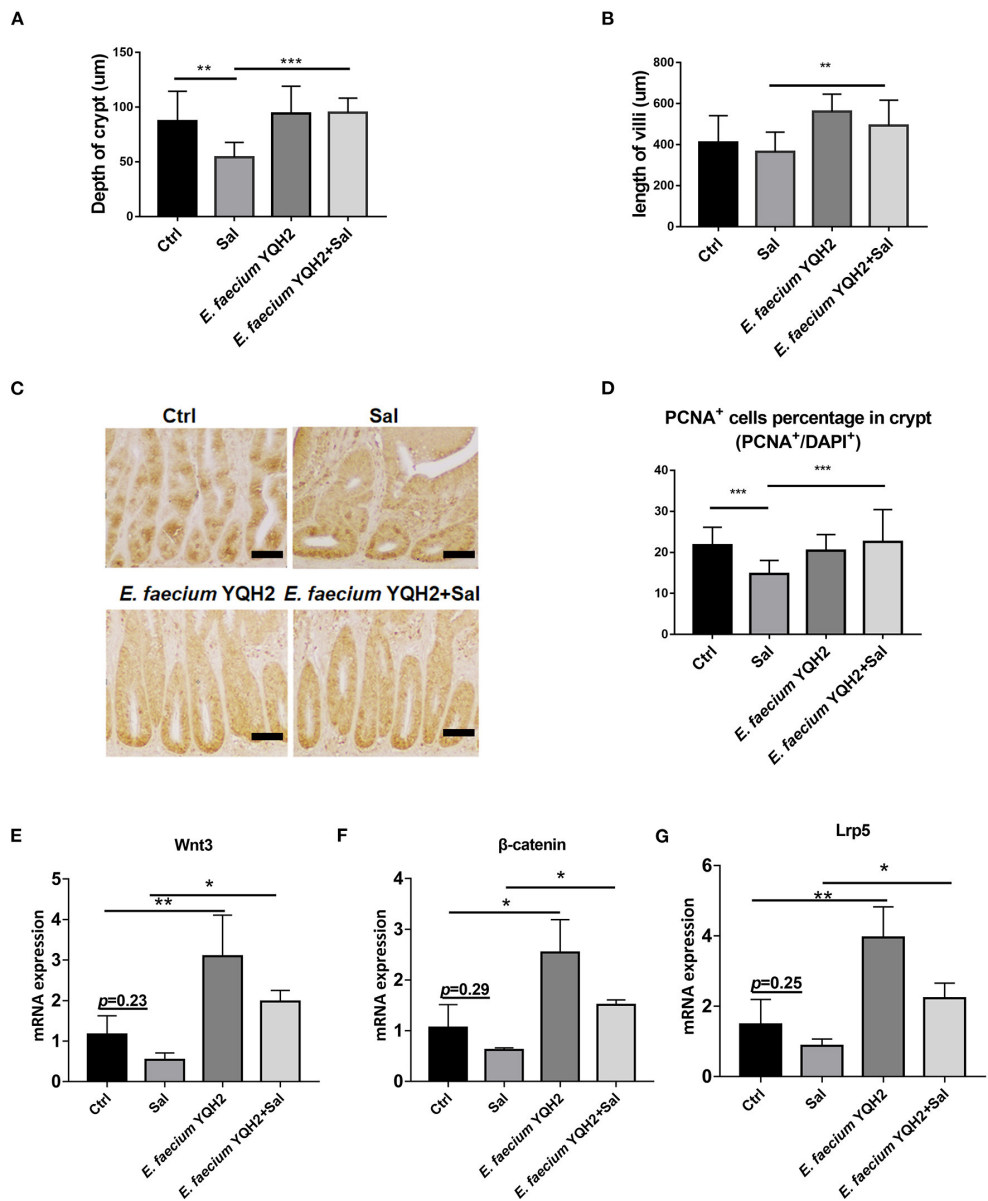
*Enterococcus faecium* YQH2 increased Wnt expression to promote steam cell proliferation. **(A,B)** The height of jejunum villus and the depth of crypt were measured in different groups, *n* = 50 (scale bars = 50 μm). **(C,D)** Immunohistochemistry staining of PCNA in ileums of chicken. The number of PCNA-positive cells in at least 50 crypts was detected (scale bars = 50 μm). **(E–G)** The expression of proliferating genes in intestinal stem cells in the ileum, including Wnt3, β-catenin, and Lrp5, was detected by real-time fluorescence quantitative PCR, *n* = 8. *p < 0.05, **p < 0.01, ***p < 0.001.

The study showed that *S. typhimurium* infection shortened the ileum length and reduced crypt depth significantly (*p* < 0.01), while these pathological changes could be ameliorated notably with *E. faecium* YQH2 (*p* < 0.001) (Figures 3A,B). This study indicated that *E. faecium* YQH2 could increase intestinal proliferation. The number of proliferating cell nuclear antigen (PCNA)–positive cells was reduced by *S. typhimurium* infection whereas *E. faecium* YQH2 reversely promoted intestinal epithelium proliferation, with increased PCNA^+^ cells (*p* < 0.001) (Figures 3C,D). In addition, *Salmonella typhimurium* could inhibit the mRNA expression of Wnt/β-catenin signaling pathway–related genes (Wnt3, β-Catenin, Lrp5), but after *Enterococcus faecium* treatment, the expression of these factors significantly increased (*p* < 0.05) (Figures 3E–G). The study further demonstrated that *E. faecium YQH2* promoted intestinal stem cell proliferation by activation of Wnt/β-catenin pathway.

### *Enterococcus faecium* YQH2 Inhibited the Activation of Hypoxia Induced by *Salmonella typhimurium*

**Figure 4:**
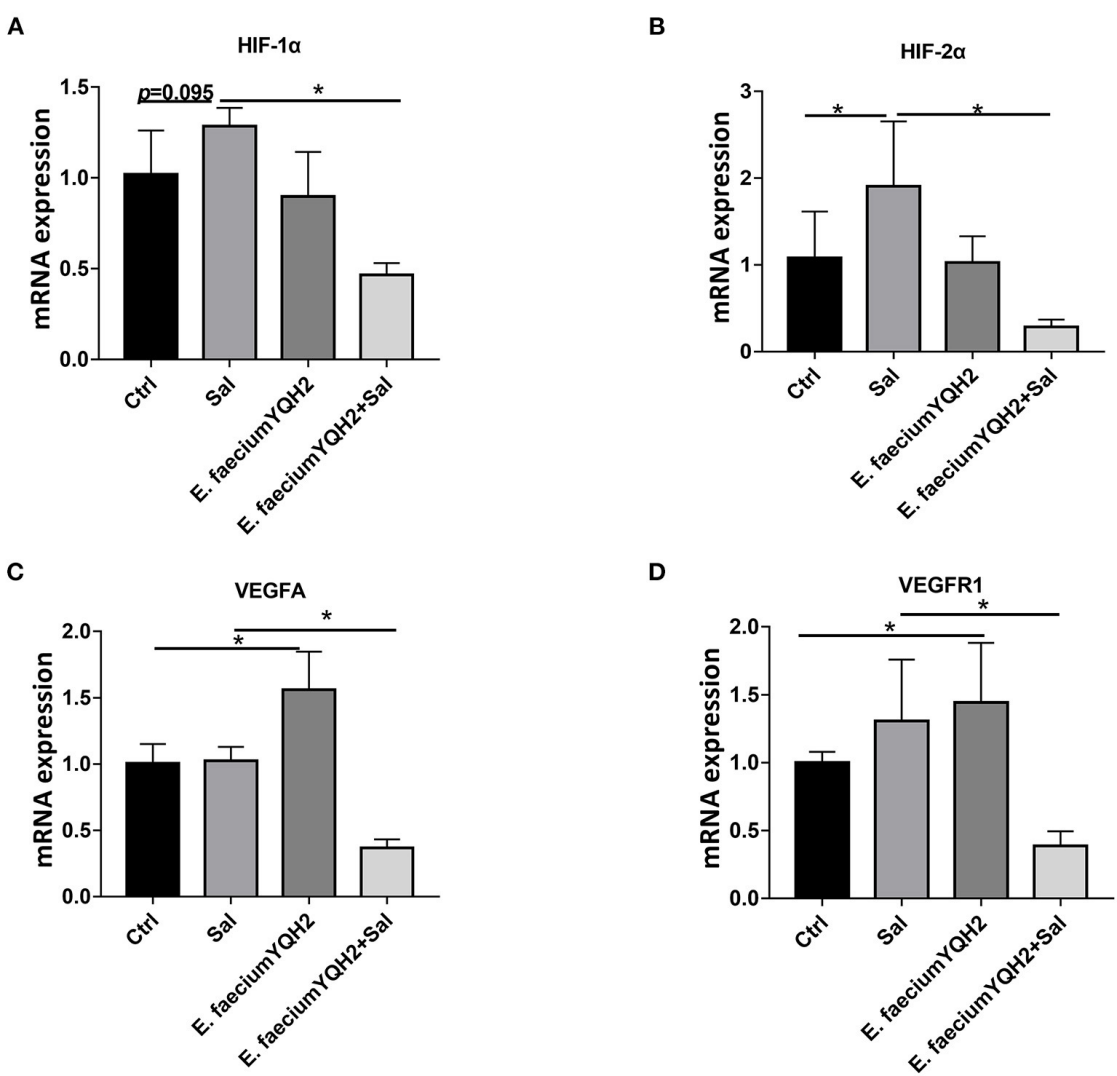
*Enterococcus faecium* YQH2 inhibited the activation of hypoxia induced by *S. typhimurium*. **(A–D)** The mRNA expressions of hypoxia-related genes in ileum was detected by real-time quantitative PCR, including HIF-1α, HIF-2α, VEGFA, and VEGFR1, *n* = 8. *p < 0.05.

HIF-1α can mediate hypoxia signals and play an important role in the physiological and pathological processes of animals. In the process of inflammation, hypoxia-inducible factors can induce the release of inflammatory factors from immune cells. Compared with PBS-treated chicken, the mRNA expression of HIF-1α in *S. typhimurium–*infected chicken have a moderate growth as the expression of HIF-2α increases significantly (*p* < 0.05) (Figures 4A,B). This result showed that hypoxia could be stimulated by *S. typhimurium* in gut. Under physiological conditions, *E. faecium* YQH2 activated the downstream target gene of hypoxia-inducible factor VEGFA and VEGFR1. However, after the infection of *S. typhimurium*, in an inflammatory state, both the expression of downstream target gene and hypoxia-inducible factor were significantly inhibited by *E. faecium* YQH2 (Figures 4C,D). Together, this study indicated that *E. faecium* YQH2 could stabilize the level of hypoxia-inducible factor and then improve the intestinal mucosa damage induced by *S. typhimurium*.

## Discussion

*Salmonella* enteritis is the result of acute intestinal inflammation caused by pathogens triggering the host's innate immune response (25). The active bacterial invasion of intestinal epithelial cells (IECs) is the key aspect of pathogenesis. When animals develop enteritis, the inflammatory factors in the intestine are usually increased (26). The rise of inflammatory factors lead to the destruction of the intestinal epithelium (27), and ultimately destroyed the intestinal mucosal barrier (28). Compared with adult chickens, chicks were more susceptible because of their underdeveloped immune system (29). Considering the limitations placed upon the use of antibiotics in poultry farming, there is an urgent need for eco-friendly probiotics for prevention of pathogenic infections. In our study, although under normal physiological conditions, the body weight of broiler chickens after YQH2 treatment with *Enterococcus faecium* did not increase, and the colonization of *Salmonella typhimurium* and the level of some inflammatory factors were significantly reduced. It was interesting that *Enterococcus faecium* YQH2 maintained the body weight of broiler chickens under *Salmonella typhimurium* infection, and reduced the colonization of pathogens and the level of inflammatory factors.

*Salmonella typhimurium* first colonize the intestinal tract, then invade and destroy the intestinal epithelial cells, and then cross the intestinal epithelial barrier to cause intestinal inflammation and even systemic infection (30, 31). This study showed that *E. faecium* YQH2 effectively reduced the colonization of *S. typhimurium*, which may be attributed to the alleviation of intestinal mucosa and liver damage. In *Enterococcus faecium* YQH2 under the conditions of *Salmonella typhimurium* infection, the reduction in the colonization of pathogens was also consistent with the reduction in the production of pro-inflammatory cytokines (such as IL-1β and TNF-α). IL-1β is an inflammatory mediator, mainly produced by monocytes, macrophages, and endothelial cells. TNF-α is a pro-inflammatory cytokine that induces and activates neutrophils and participates in local inflammation (32, 33). It is reported that Toll-like receptors play an important role in the innate immune response, especially in the initial interaction between infected microorganisms and phagocytes, such as macrophages (34). TLR4 and MyD88 are important for the chicken after being infected with *S. typhimurium* (35). Through this study, we further found that the mRNA expression of TLR4 and NF-κB in chickens infected with *S. typhimurium* was distinctly increased, which then resulted in the terminal activation of inflammatory regulators NF-κB.

During *S. typhimurium* infection, intestinal inflammation is always accompanied with intestinal epithelial cell damage. Therefore, the proliferation of intestinal stem cells and replacement of damaged intestinal epithelial cells after injury are essential for intestinal wound healing (36, 37). Wnt is known to regulate intestinal stem cells and β-catenin is at the downstream end of Wnt pathway (38). Previous studies reported that *S. typhimurium* secretes AvrA effector protein into host cells, therefore preventing the ubiquitination and degradation of β-catenin, which leads to the activation of Wnt/β-catenin pathway, accompanied with over-proliferation of intestinal cells and crypt hyperplasia in mice (39). The similar activation of Wnt/β-catenin pathway was also found in chickens with *S. pullorum* infection, as well as in mice with *S. typhimurium* infection (40, 41). However, AvrA and SopB secreted by *Salmonella* activated Wnt signaling in intestinal M cells and crypt-localized epithelial cells, but inhibited Wnt signaling in intestinal epithelial cells (42). In this study, we observed that *S. typhimurium* infection in chicken caused inhibition of Wnt/β-catenin pathway, which was also verified with the reduced crypt depth and villi length, as well as reduced number of PCNA^+^ cells in crypt. This damage to intestinal mucosa has attributed to the serious intestinal inflammation under *S. typhimurium* infection, which surpassed the repair process of intestinal epithelial cells. However, *E. faecium* YQH2 could reversely induce the activation of Wnt/β-catenin pathway to support the protectory effect.

Recent study demonstrated that the state of low oxygen concentrations in the intestinal mucosa was also closely related to intestinal inflammation (43). Hypoxia-inducible factor-1 (HIF-1) is the core factor in regulating the hypoxia stress response and mediating the physiological behavior of hypoxic cells, HIF-1α also inhibits the activation of β-catenin that is mediated by hARD1, then downregulates expression of Wnt in gastric cancer cells or osteoblasts (44). It has been demonstrated that HIF-1α plays a significant role in the inhibition of β-catenin by blocking interaction of transcription factor 4 with β-catenin (45). In our study, we observed that *S. typhimurium* infection induced the high expression of HIF which inhibited the activation Wnt/β-catenin expression in intestine. However, *E. faecium* YQH2 treatment has significantly decreased the level of HIF-1α under *S. typhimurium* infection and promoted the proliferation of intestinal stem cells. A previous study also demonstrated that increased expression of HIF-1α would activate NF-κB signaling pathway through TLR4 and increase inflammatory response (46). This relationship between HIF and NF-κB could also explain the protectory effect of *E. faecium* YQH2 on maintaining the normal levels of HIF and NF-κB.

In conclusion, the results suggested that *E. faecium* YQH2 improved the intestinal mucosal damage caused by *S. typhimurium* in chicken. *E. faecium* YQH2 reduced the over-action of HIF, which then stimulated the Wnt/β-catenin pathway to promote the proliferation of intestinal epithelial cells, as well as reduced the intestinal inflammation level through TLR4/NF-κB pathway.

## Data Availability Statement

The raw data supporting the conclusions of this article will be made available by the authors, without undue reservation.

## Ethics Statement

The animal study was reviewed and approved by the Institutional Animal Care and Use Committee (IACUC) of Nanjing Agricultural University. Written informed consent was obtained from the owners for the participation of their animals in this study.

## Author Contributions

HZ, MW, and JZ were responsible for performing the experiments and data analysis. JJ and RS were responsible for data verification and article polish. QY was responsible for the conception and design of the study and final approval of the version submitted. All authors contributed to the article and approved the submitted version.

## Funding

This study was supported by the National Key R&D Program of China (2018YFE0127300), the State Key Laboratory of Veterinary Etiological Biology, Lanzhou Veterinary Research Institute, Chinese Academy of Agricultural Sciences (SKLVEB2019KFKT004), Fundamental Research Funds for the Central Universities (JCQY201906), National Natural Science Foundation of China (31972631), and a project funded by the Priority Academic Program Development of Jiangsu Higher Education Institutions (PAPD).

## Conflict of Interest

The authors declare that the research was conducted in the absence of any commercial or financial relationships that could be construed as a potential conflict of interest.

## Publisher's Note

All claims expressed in this article are solely those of the authors and do not necessarily represent those of their affiliated organizations, or those of the publisher, the editors and the reviewers. Any product that may be evaluated in this article, or claim that may be made by its manufacturer, is not guaranteed or endorsed by the publisher.
